# Novel application of *In Vivo* Micro-Optical Coherence Tomography to assess Cornea scarring in an Animal Model

**DOI:** 10.1038/s41598-018-29761-4

**Published:** 2018-07-31

**Authors:** Marcus Ang, Kavya Devarajan, Suchandrima Das, Gary H. F. Yam, Hla Mynt Htoon, Si Chen, Xinyu Liu, Linbo Liu, Michael Girard, Jodhbir S. Mehta

**Affiliations:** 1 Singapore National Eye Centre, Singapore, Singapore; 2 Singapore Eye Research Institute, Singapore, Singapore; 3 Department of Ophthalmology and Visual Science, Duke-NUS Graduate Medical School, Singapore, Singapore; 4 Moorfields Eye Hospital, London, United Kingdom; 5 School of Electrical & Electronic Engineering and School of Chemical & Biomedical Engineering, Nanyang Technological University, Singapore, Singapore; 6 Ophthalmic Engineering and Innovation Laboratory, Department of Biomedical Engineering, Faculty of Engineering, National University of Singapore, Singapore, Singapore

## Abstract

This pilot study uses a micro-optical coherence tomography (micro-OCT) system with ~1 μm axial resolution specifically to image the cornea and corneal scars *in vivo*. We used an established murine corneal scar model by irregular phototherapeutic keratectomy in ten C57BL/6 mice, with serial imaging using the micro-OCT and compared to anterior segment (AS-OCT) (RTvue, Optovue, Fremont, CA) before and after scar induction. Main outcome was agreement between the AS-OCT and micro-OCT using Bland-Altman plots (95% limits of agreement, LoA).We analysed 10 control eyes and 10 eyes with corneal scars and found that there was good agreement between AS-OCT and micro-OCT (P > 0.05) LOA: lower limit −14 µm (95% CI: −19 to −8.8 µm) upper limit 23 µm (95% CI: 18 to 28.5 µm) in terms of central corneal thickness. There was also good agreement between AS-OCT and micro-OCT in terms of corneal scar measurements (P > 0.5; correlation coefficient >0.99) LOA lower limit −2.1 µm (95% CI: −2.8 to −1.5 µm); upper limit 1.8 µm (95% CI: 1.1 to 2.4 µm). Our pilot study suggests that this novel *in vivo* micro-OCT imaging technique was able to measure central corneal thickness and scar thickness in agreement with current AS-OCT techniques.

## Introduction

Optical coherence tomography (OCT) uses low-coherence interferometry to enable non-contact, *in vivo* high-resolution imaging of the cornea^1^. However, most commercial anterior segment OCT (AS-OCT) systems are only able to achieve up to 5 μm axial resolution^2^ and thus, not able to differentiate layers within the cornea^3^. We have previously described a micro-OCT that uses broadband light to produce images of the cornea with up to 1–2 μm spatial resolution^4–6^. The micro-OCT was able to image corneal endothelial cells^4,5^ with a much higher resolution compared to a spectral domain OCT^7^.

Corneal scarring secondary to various insults may cause permanent visual impairment, where corneal transplantation may be the only way to restore vision^8^. As most corneal lesions or scars do not exist throughout the cornea, lamellar replacement of the diseased corneal layers^9^, may provide better tectonic outcomes^10^, or faster visual rehabilitation^11–13^. Thus, delineation of the cornea is important to allow for targeted treatment – while real-time AS-OCT guidance has now become possible in our surgical practice^14^. However, current AS-OCT systems have limited resolution, which are only able to detect gross changes to the cornea without detailed imaging of corneal layers, especially in the presence of corneal scars or intrastromal lesions.

Therefore, we performed a pilot study using a superluminescent diode array based micro-OCT system with ~1 μm axial resolution to detect diseased corneal layers, i.e. even in the presence of corneal scarring. Here, we compared this *in vivo* micro-OCT to a commercially available AS-OCT system in an established animal model to assess normal and scarred corneas.

## Results

**Figure 1:**
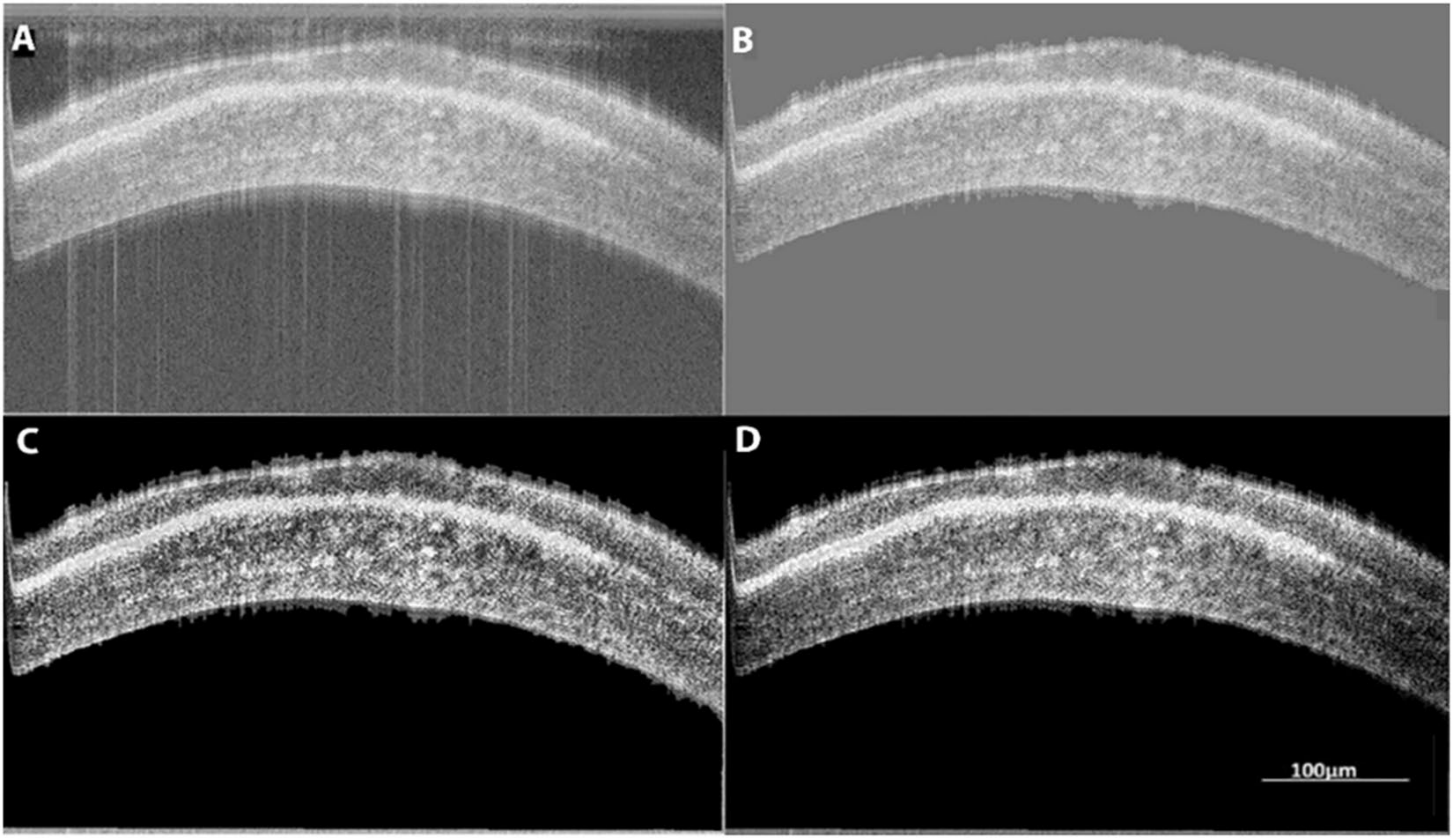
Example of micro-OCT Imaging of corneal scar with image processing for micro-OCT image enhancement. (**A**) Raw OCT image with noise and distortion. (**B**) Background noise and edge distortions removed using filters. (**C**) Image compensated for light attenuation and contrast enhancement. (**D**) Post-processing for improved visibility of tissue layers.

**Figure 2:**
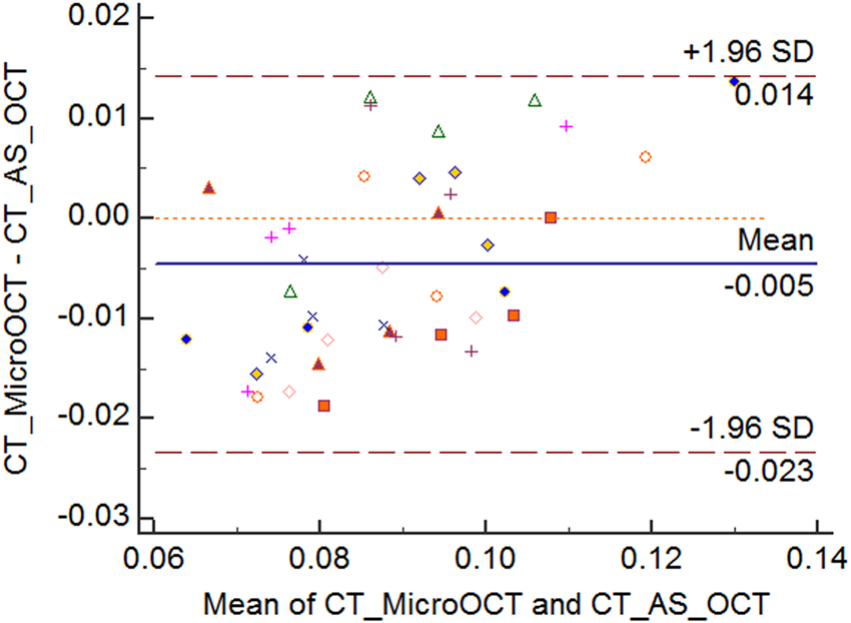
Bland-Altman plot comparing corneal thickness measurements between AS-OCT and Micro-OCT. Each marker represents one mouse cornea of which the central corneal thickness was measured during the follow-up imaging. Solid line = mean of the difference. Short dashed line = reference zero. Long dashed line = upper and lower 95% limits of agreement (mean +1.96 SD, mean −1.96 SD). SD = standard deviation of the mean difference.

The analysis was done across all corneal samples (10 control and 10 injured). First, we compared normal corneal thickness measurements between established AS-OCT and micro-OCT to validate that the novel micro-OCT was comparable – Fig. 1. We found that the overall mean at baseline central corneal thickness (CCT) was 94.8 ± 4.0 µm using the AS-OCT and 91.4 ± 6.0 µm using the micro-OCT. There were no significant differences in mean CCT measured under AS-OCT and micro-OCT (Table 1). There was a good agreement between these two imaging techniques (P > 0.05) as the LOA were ranged from a lower limit of −14 µm (95% CI: −19 to −8.8 µm) to upper limit of 23 µm (95% CI: 18 to 28.5 µm) – Fig. 2.

**Table 1:** Comparison of *in vivo* central corneal thickness measurements (milimeters) between anterior segment optical coherence tomography and micro-optical coherence tomography.

Phase – Corneal thickness	Micro-OCT Mean Thickness		AS-OCT Mean Thickness		Mean Difference (Bias)		P-value*	95% Confidence Interval - Bias	95% CI of LOA	
	Mean (N = 10)	S.D	Mean (N = 10)	S.D	Mean (N = 10)	S.D			Lower LOA	Upper LOA
Baseline	0.0914	0.006	0.0948	0.004	0.003	0.006	0.1456 (>0.05)	(−0.0014, 0.008)	(−0.01865, −0.0013) µ = −0.009	(0.008, 0.025) µ = −0.016
Week 1	0.0802	0.0072	0.0854	0.0051	0.005	0.011	0.1738 (>0.05)	(−0.0028, 0.0133)	(−0.0311, −0.00255) µ = −0.016	(0.0131, 0.041) µ = −0.027
Week 2	0.0731	0.004	0.0833	0.0037	0.010	0.006	0.0012 (<0.05)	(0.005, 0.0151)	(−0.0121, 0.00541) µ = −0.003	(0.0150, 0.032) µ = −0.023
Week 4	0.1009	0.006	0.1003	0.004	0.0006	0.0103	0.8564 (>0.05)	(−0.007, 0.006)	(−0.0338, −0.00775) µ = −0.0208	(0.006, 0.032) µ = −0.019

**Figure 3:**
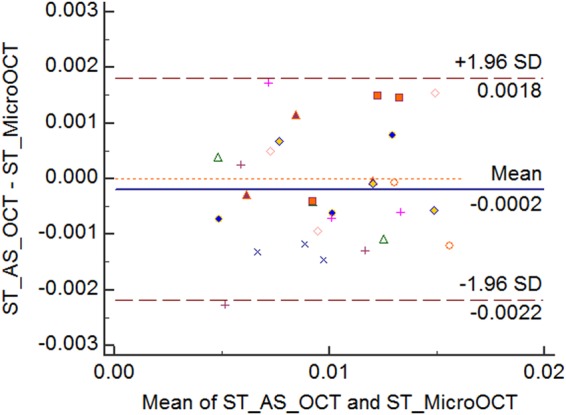
Bland-Altman plot comparing scar thickness measurements between AS-OCT and micro-OCT. Each label represents one mouse cornea of which the scar thickness was measured during the follow-up imaging. Solid line = mean of the difference. Short dashed line = reference zero. Long dashed line = upper and lower 95% limits of agreement (mean +1.96 SD, mean −1.96 SD). SD = standard deviation of the mean difference.

Next, we compared the ability of the micro-OCT to measure the corneal scar thickness as compared to the AS-OCT. The mean scar thickness was 6.9 ± 3.3 µm measured by AS-OCT and 6.9 ± 3.0 µm by micro-OCT wherein no significant difference was observed between both the imaging modalities (Table 2). The central scar was developed to the greatest thickness at week 2 and subsequently reduced by week 4. There was a good agreement between these two imaging techniques (P > 0.5; correlation coefficient >0.99) with the LOA ranged from a lower limit of −2.1 µm (95% CI: −2.8 to −1.5 µm) to upper limit of 1.8 µm (95% CI: 1.1 to 2.4 µm) – Fig. 3.

**Table 2:** Comparison of *in vivo* central corneal scar thickness measurements (milimeters) between anterior segment optical coherence tomography and micro-optical coherence tomography.

Phase- Scar thickness	Micro-OCT Mean Thickness		AS-OCT Mean Thickness		Mean Difference (Bias)		P-value*	95% Confidence Interval - Bias	95% CI of LOA	
	Mean (N = 10)	S.D	Mean (N = 10)	S.D	Mean (N = 10)	S.D			Lower LOA	Upper LOA
Week 2	0.0132	0.003	0.0129	0.0029	0.0002	0.001	0.5108 (>0.05)	(−0.001, 0.0005)	(−0.0039, −0.001) µ = −0.0024	(0.0005, 0.003) µ = 0.0019
Week 3	0.0099	0.0024	0.0098	0.003	0.0001	0.0008	0.6956 (>0.05)	(−0.0007, 0.0005)	(−0.0029, −0.0007) µ = −0.0018	(0.0004, 0.0026) µ = 0.0015
Week 4	0.0069	0.003	0.0067	0.0033	0.0002	0.001	0.56 (>0.05)	(−0.001, 0.0005)	(−0.0038, 0.0009) µ = −0.0024	(0.0005, 0.0033) µ = 0.0019

**Figure 4:**
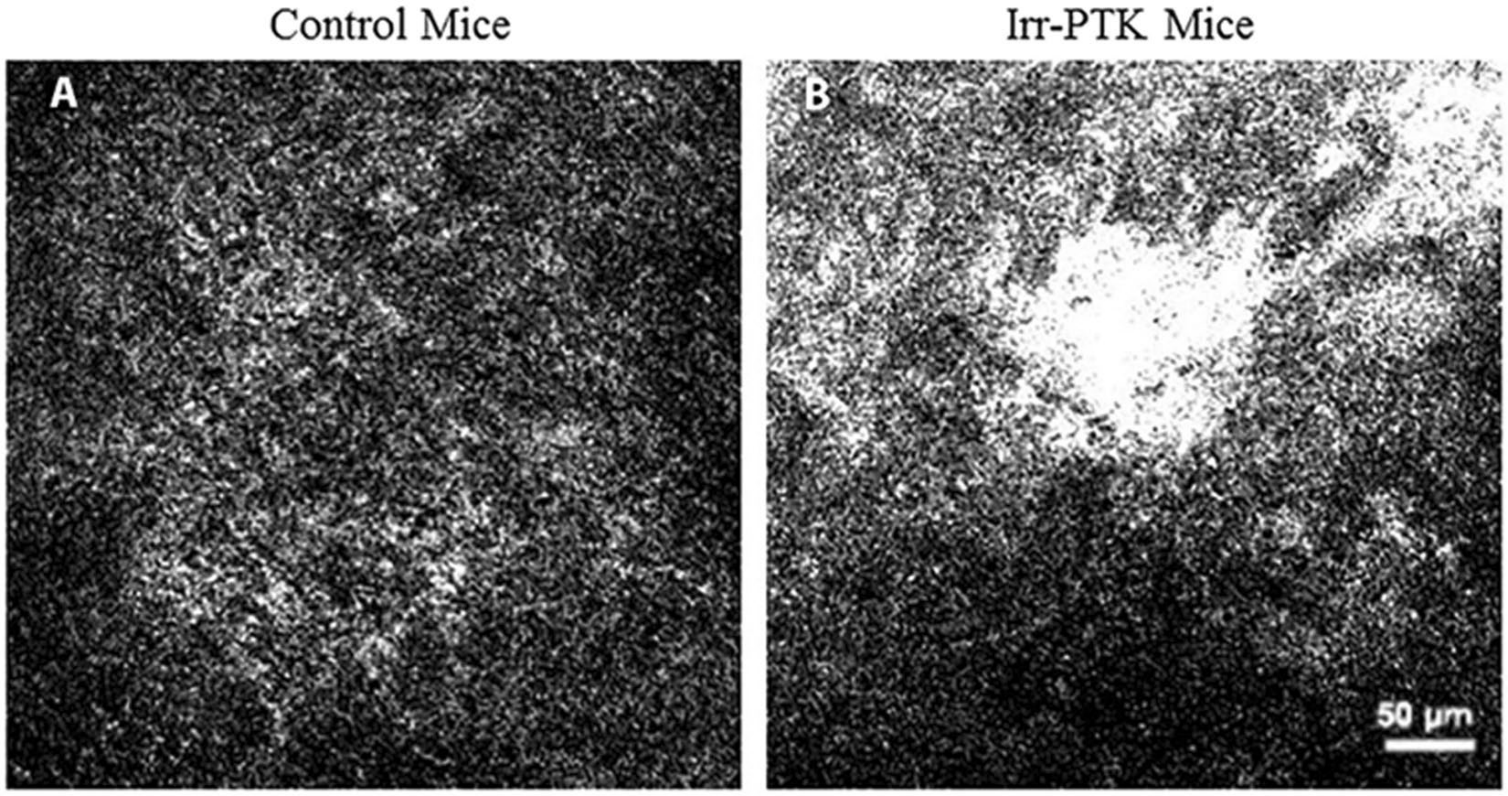
Examples of *in vivo* confocal microscopy images in (**A**) area of normal cornea in the control eyes showing normal stromal cells with dark background; (**B**) area of corneal scarring exhibiting strong stromal reaction due to the presence of active stromal fibroblasts depicted by the hyper-reflective region. Scale bar is 50 µm.

**Figure 5:**
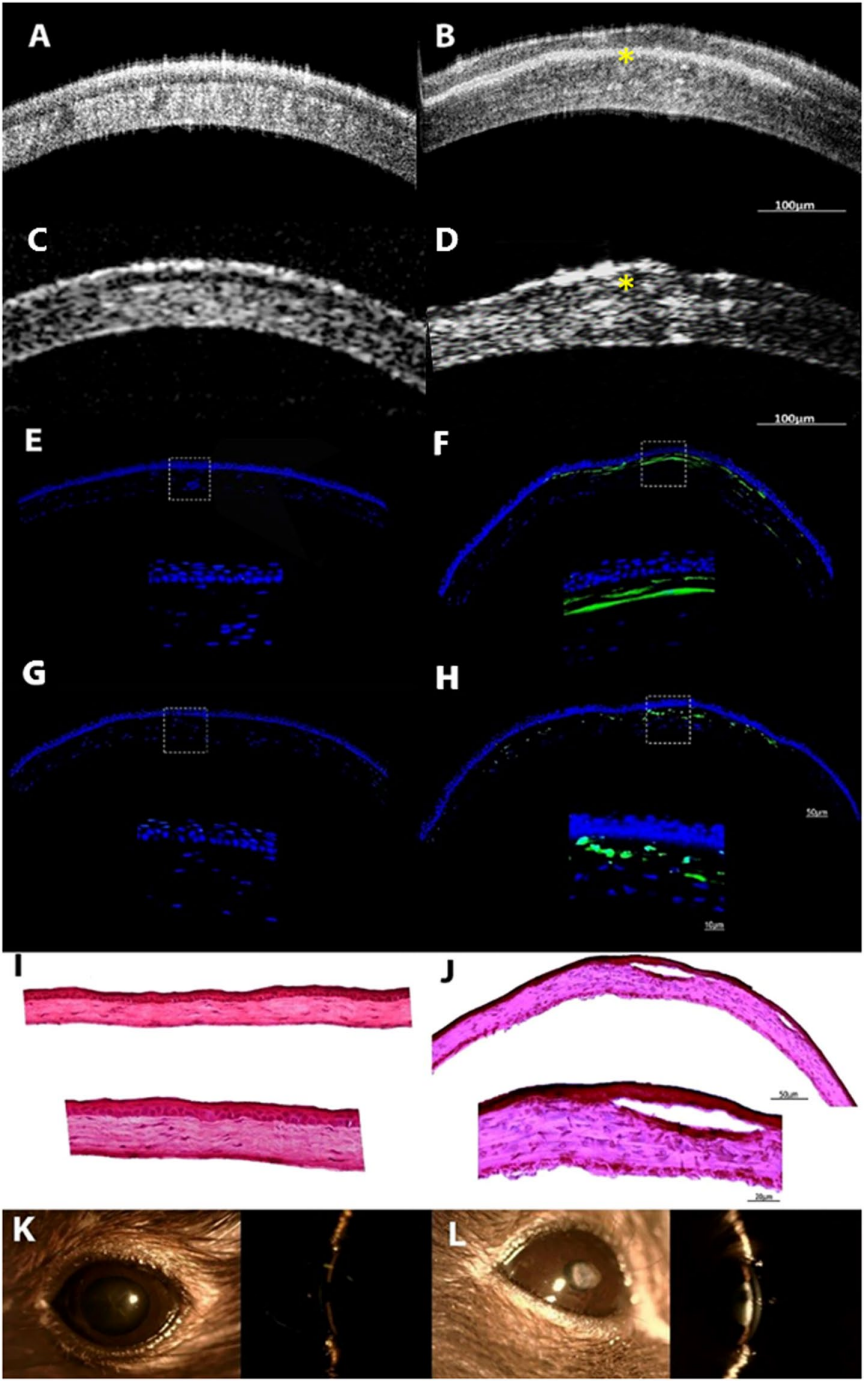
Representative micro-OCT of control and stromal scarred cornea comparing AS-OCT, histology and slit lamp photography. (**A**–**D**) Example images from micro-OCT (**A**,**B**) and conventional AS-OCT imaging (**C**,**D**); Figures **A** and **C** represent the control cornea; whereas **B** and **D** represent the scarred cornea where the yellow asterisk is marked to identify the wound. The micro-OCT is able to show the corneal scar with much higher definition depicting changes to lamellar layers i.e. the anterior stroma and epithelium (**A**,**B**). (**E**–**H**) Immunohistochemical staining for alpha smooth muscle actin (α-SMA) and for fibronectin (FN) and in mice corneas at three weeks after injury. (**E**) Example of a normal control cornea showing absence of α-SMA. (**F**) Example of scarred cornea showed intense α-SMA staining in the sub-epithelial stroma, indicating haze development corresponding to the scar seen in the micro-OCT images. (**G**) Example of a normal control cornea without scarring showing the absence of FN staining. (**H**) Example of a scarred cornea with distinct FN signal in the central stromal region Cell nuclei were stained with FN, α-SMA signals were in green fluorescence. (**I**,**J**) Hemotoxylin and Eosin (H&E) staining of (**I**) Control cornea showing no evidence of scarring or inflammation. (**J**) Injured cornea showing the area of scar with increased inflammatory cell infiltration and epithelial disorganization which corresponded to the area of scar detected by the micro-OCT. (**K**,**L**) Examples of slit-lamp photographs showing normal (**K**) and scarred (**L**) corneal, slit-beam to demonstrate the scars are mainly in the anterior stromal aspects.

The micro-OCT successfully identified the area of corneal scarring as confirmed by corresponding *in vivo* confocal microscopy imaging (Fig. 4) and histology (Fig. 5). Hematoxylin and eosin histochemistry in scarred corneas at three weeks confirmed corresponding scarring with increased inflammatory cell infiltration in the corneal stroma, and distinct epithelial disorganization. We also used the micro-OCT to identify the areas of active scarring, confirmed by the presence of myofibroblasts and fibroblasts using immunostaining for alpha smooth muscle actin (α-SMA) and fibronectin – Fig. 5. The scar detected by the micro-OCT corresponded to the areas of α-SMA staining and strong fibronectin expression compared to the control, normal corneas.

## Discussion

Our study using a novel *in vivo* micro-OCT imaging technique suggested that the measurement of central corneal thickness and scar thickness were generally of good agreement with AS-OCT images, with at least 95% of measurements and differences within the 95% prediction intervals and 95% LOA, respectively. We found that the images acquired were able to discern the five basic corneal layers. When imaging the irr-PTK injured eyes, the scar tissue was also detected prominently. For example, the mean central corneal (80.2 ± 11.28 μm) and scar thickness (9.9 ± 2.28 μm) in mice cornea determined by the micro-OCT showed good agreement with immunofluorescent histology measurements of (82 ± 5.5 μm) CCT and (9.2 ± 3.5 μm) scar obtained at Week 2 follow-up. The resolution of distinction of the corneal layers in the processed micro-OCT acquired images, are comparable to the histology results, suggesting the potential for OCT to produce high resolution images of specific corneal layers non-invasively. When comparing the micro-OCT images with the AS-OCT imaging modality, the former performs better than AS-OCT in prominently displaying the scar distribution and intensity.

However, it is important to note the limitations of the *in vivo* micro-OCT used in this pilot study. One of the main problems encountered was the widespread presence of motion artefacts. The reason behind this could be attributed to the heart beat rate (310–840 beats per minute) of the mice used herein, which was much higher than the image acquisition speed of 60 frames per second. In view of this, future outlook for this area of study may involve improving the image acquisition speed. Moreover, the image acquisition for the micro-OCT is longer (estimated 1–2 minutes per image) compared to IVCM (15–30 seconds) and AS-OCT (5 seconds). Nonetheless, we obtained promising results, where this non-invasive imaging technique could produce images with quality comparable to that of *ex vivo* histology, and with more detail as compared to the current AS-OCT. Further improvements in the image processing technique and image acquisition speed, may allow better results for human eyes^15,16^.

The recent advances in OCT technology now allows, not only higher resolution imaging of the cornea^17^, but also additional information such as en face reconstruction for the cornea and ocular surface^18^, or even delineating vascular flow within the cornea^19–21^. High-resolution OCT is currently able to image corneal layers i.e. epithelium, Bowman’s layer, stroma, Descemet membrane, endothelium - and even the ocular surface tear film^22^. However, we have described a micro-OCT system that is able to provide more details similar to *in vivo* microscopy imaging, which may have a potential role in corneal pathologies that specifically require more information such as surgical planning in corneal dystrophies^23^, atypical corneal infections requiring microscopic detail of the pathogen^24^, or assessing the cornea after refractive surgery where high definition stromal layer assessment is required^25,26^.

In summary, despite the recognized limitations of this *in vivo* micro-OCT system, we provide promising early results from an animal study that suggests high resolution images with histology like detail are obtained in both normal and scarred corneas. Technical improvements such as developing a swept source micro-OCT system with higher A-line speed (up to 4 MHz)^27^, or introduction of a line scan camera with full-range OCT imaging^28^, may improve the image quality even further. If successful, the increased amount of detail in corneal structure analysis could have clinical applications in assessing disease within corneal layers or planning for selective corneal replacement. Further studies for human corneal imaging use *in vivo* would be required to establish this promising micro-OCT system in the future.

## Methods

This study was approved by the Institutional Animal Care and Use Committee of Singapore Health Services and followed the guidelines in the use of Animals in Ophthalmic and Vision Research. We used a murine corneal scar model by irregular phototherapeutic keratectomy as previously described^13^. Ten C57BL/6 mice were anaesthetized by an intraperitoneal injection of ketamine hydrochloride (20 mg/kg body weight) and xylazine hydrochloride (2 mg/kg body weight), with only one eye treated under topical anesthesia (topical 1% lignocaine hydrochloride). Briefly, a standard 2 mm diameter central corneal wound was made using #64 Beaver blade and denuded of epithelium. Irregular PTK (irrPTK) was then performed to the ablation zone by firing 105 laser pulses (ablation depth ~10 μm) with an excimer laser. A fine mesh screen was positioned in the path of laser after firing 50% of the pulses performed with PTK. Discomfort was relieved by subcutaneous injection of Buprenorphine, 0.05 mg/kg body weight, twice daily for 1 day. Tobramycin ointment was applied twice daily for 2 days to prevent infection. Both injured and fellow control eyes were imaged under micro-OCT, slit lamp biomicroscopy, *in vivo* confocal microscopy and standard OCT (described in detail below) at day 3 before and 7, 14, 21, 28 days after scar induction. The mice were sacrificed at the 5^th^ week.

### Micro-Optical Coherence Tomography Imaging

The micro-OCT essentially uses light scattered elastically from within tissue in three dimensions and uses the electric field amplitude and the optical delay of light returned from the sample to measure the depth of the tissue. In this study, the micro-OCT system was modified from that described previously^29^, with significant improvements to axial and lateral resolution. The high-bandwidth (~165 nm centered at 830) and short coherence length light, is obtained by use of a superluminescent diode source (T-850, Superlum, Ireland), allowing axial resolution of 2.5 μm in air. The system includes an interferometer while the reference and sample arms intersect at the beam-splitter. A lateral resolution of 2.5 μm (10X objective lens) and 1.3 μm (20X objective lens) is achieved. Effective power incident on sample was kept less than 2 mW. The galvanometer scanning motors were controlled using customizable software, in correspondence with the spectral-data from a scan camera (E2V, AViiVA EM4). The line rate (1024 per frame), frame rate (60 per second) and the scan geometry (3.5 mm × 3.5 mm) were used. Three dimensional images (3.5 mm × 3.5 mm × 1 mm, W × H × D) were obtained with an acquisition time of less than 3 minutes per 3D image.

### Optical Coherence Tomography Image Enhancement and Data collection

Current image quality from standard OCT is greatly hampered by the presence of shadow artifacts and poor tissue visibility in the deeper layers - due to signal attenuation, whereby signal strength diminishes as a function of tissue depth. Moreover, the animal model for this experiment, mice, have a very high heart rate of 310–840 beats per minute, which being much higher than the frame rate results in noise and distortion in the captured image. Filters were applied to remove the background noise and edge distortions from the images using MATLAB R2017a (The MathWorks, Inc., Natick, Massachusetts, United States). Further, the captured micro-OCT images were improved using compensation algorithms to compensate for light attenuation and contrast enhancement with post-processing software^15^, used to remove the shadow artefacts from dense structures and improve the visibility of the deep tissue layers, as previously described – Fig. 1^16^. The post-processed images were then used to obtain information on the corneal, epithelial and the scar thickness (in case of the injured samples). All measurements were done using ImageJ and each measurement was computed as the average of 10 measurements taken across the sample.

### Anterior segment evaluation and histology

We performed serial slit-lamp microscopy (FS-3V Zoom Photo Slit Lamp, Nikon, Tokyo, Japan) and AS-OCT (RTvue, Optovue, Fremont, CA) to compare corneal measurements between the micro-OCT and AS-OCT. *In vivo* confocal microscopy (Heidelberg Engineering GmbH, Heidelberg, Germany) was also performed for corneal scars *in vivo*. After sacrifice, the murine corneas were extracted and fixed in 10% formaldehyde (Sigma Aldrich, St. Louis, MO, USA) for 60 min and PBS washes. They were dehydrated and processed for paraffin embedding, with sections (5 μm thick), haematoxylin and eosin (H&E) staining - using Mayer’s hematoxylin (Sigma Aldrich, USA), then differentiated with acid alcohol, extensively washed before stain with Eosin solution (Sigma Aldrich, USA) for another 2 minutes, washed, and dehydrated. The section was mounted with Permount reagent (Fisher) and viewed under light microscopy (Nikon C2 confocal microscope). Immunofluorescence staining for scar tissue markers was performed using Mouse On Mouse (MOM) Immunodetection kit (Vector Laboratories Inc., Burlingame, CA, US) with mouse monoclonal antibody against alpha smooth muscle actin (α-SMA) (DAKO M0851; 1:200 dilution) and mouse monoclonal antibody against fibronectin (FN) (Millipore MAB1940; 1:200 dilution; Temecula CA, US). After staining, the samples were mounted with Vectashield containing DAPI (Vector Laboratories) and viewed under fluorescence microscopy (ZEISS Axio Observer.Z1, Oberkochen, Germany).

### Statistical analysis

Mean differences in corneal and scar measurements (mean ± standard deviation, SD) between the two machines were assessed using a paired t-test, after tests for normality. Agreements between the AS-OCT and µ-OCT machines were also described using the Bland-Altman, with 95% limits of agreement (LoA). Bland Altman analysis was performed with MedCalc Version 17.1. Intra-Class Correlation Coefficients were computed for inter-machine reliability of data. All other statistical tests were performed with IBM Corp. Released 2011 IBM SPSS Statistics for Windows, Version 20.0. Armonk, NY: IBM Corp.; Statistical significance was considered p < 0.05.

### Data availability

The data that support the findings of this study are available from the corresponding author.

## References

[1] Ang M (2012). Anterior segment optical coherence tomography study of the cornea and anterior segment in adult ethnic South Asian Indian eyes. Invest Ophthalmol Vis Sci.

[2] Ang, M. *et al*. Anterior segment optical coherence tomography. *Prog Retin Eye Res.* pii: S1350-9462(17)30085-X, 10.1016/j.preteyeres.2018.04.002. [Epub ahead of print] Review. PMID:29635068 (2018). 10.1016/j.preteyeres.2018.04.002 29635068

[3] Girard MJ, Strouthidis NG, Ethier CR, Mari JM (2011). Shadow removal and contrast enhancement in optical coherence tomography images of the human optic nerve head. Invest Ophthalmol Vis Sci.

[4] Liu L (2013). Method for quantitative study of airway functional microanatomy using micro-optical coherence tomography. Plos One.

[5] Liu L (2011). Imaging the subcellular structure of human coronary atherosclerosis using micro-optical coherence tomography. Nature Medicine.

[6] Liu L (2014). An autoregulatory mechanism governing mucociliary transport is sensitive to mucus load. American journal of respiratory cell and molecular biology.

[7] Liu X, Chen S, Cui D, Yu X, Liu L (2015). Spectral estimation optical coherence tomography for axial super-resolution. Optics Express.

[8] Tan DT, Dart JK, Holland EJ, Kinoshita S (2012). Corneal transplantation. Lancet.

[9] Ang M, Mehta JS, Arundhati A, Tan DT (2009). Anterior lamellar keratoplasty over penetrating keratoplasty for optical, therapeutic, and tectonic indications: a case series. Am J Ophthalmol.

[10] Ang M, Mehta JS, Sng CC, Htoon HM, Tan DT (2012). Indications, outcomes, and risk factors for failure in tectonic keratoplasty. Ophthalmology.

[11] Tan D, Ang M, Arundhati A, Khor WB (2015). Development of Selective Lamellar Keratoplasty within an Asian Corneal Transplant Program: The Singapore Corneal Transplant Study (An American Ophthalmological Society Thesis). Trans Am Ophthalmol Soc.

[12] Ang, M. & Mehta, J. S. Deep anterior lamellar keratoplasty as an alternative to penetrating keratoplasty. *Ophthalmology* **118**, 2306–2307, author reply 2307 (2011). 10.1016/j.ophtha.2011.07.025 22047896

[13] Ang M, Mohamed-Noriega K, Mehta JS, Tan D (2013). Deep anterior lamellar keratoplasty: surgical techniques, challenges, and management of intraoperative complications. Int Ophthalmol Clin.

[14] De Benito-Llopis L, Mehta JS, Angunawela RI, Ang M, Tan DT (2014). Intraoperative anterior segment optical coherence tomography: a novel assessment tool during deep anterior lamellar keratoplasty. Am J Ophthalmol.

[15] Girard MJ (2015). Enhancement of Corneal Visibility in Optical Coherence Tomography Images Using Corneal Adaptive Compensation. Transl Vis Sci Technol.

[16] Chung CW (2016). Enhancement of Corneal Visibility in Optical Coherence Tomography Images with Corneal Opacification. Transl Vis Sci Technol.

[17] Ang M (2018). Optical coherence tomography angiography: a review of current and future clinical applications. Graefes Arch Clin Exp Ophthalmol.

[18] Ang M (2016). En face optical coherence tomography angiography for corneal neovascularisation. Br J Ophthalmol.

[19] Ang M (2015). Optical Coherence Tomography Angiography for Anterior Segment Vasculature Imaging. Ophthalmology.

[20] Ang M (2016). Optical coherence tomography angiography and indocyanine green angiography for corneal vascularisation. Br J Ophthalmol.

[21] Cai Y, A. D. Barrio JL, Wilkins MR, Ang M (2017). Serial optical coherence tomography angiography for corneal vascularization. Graefes Arch Clin Exp Ophthalmol.

[22] Moutsouris K (2011). Optical coherence tomography, Scheimpflug imaging, and slit-lamp biomicroscopy in the early detection of graft detachment after Descemet membrane endothelial keratoplasty. Cornea.

[23] Siebelmann, S. *et al*. Anterior segment optical coherence tomography for the diagnosis of corneal dystrophies according to the IC3D classification. *Surv Ophthalmol* (2017). 10.1016/j.survophthal.2017.08.001 28801092

[24] Ang M, Mehta JS, Mantoo S, Tan D (2009). Deep anterior lamellar keratoplasty to treat microsporidial stromal keratitis. Cornea.

[25] Rosas Salaroli CH, Li Y, Huang D (2009). High-resolution optical coherence tomography visualization of LASIK flap displacement. J Cataract Refract Surg.

[26] Ang M (2014). Refractive lenticule extraction: transition and comparison of 3 surgical techniques. J Cataract Refract Surg.

[27] Fechtig DJ, Schmoll T, Grajciar B, Drexler W, Leitgeb RA (2014). Line-field parallel swept source interferometric imaging at up to 1 MHz. Opt. Lett..

[28] Sarunic M, Choma MA, Yang C, Izatt JA (2005). Instantaneous complex conjugate resolved spectral domain and swept-source OCT using 3 × 3 fiber couplers. Optics Express.

[29] Ang M (2016). Evaluation of a Micro-Optical Coherence Tomography for the Corneal Endothelium in an Animal Model. Sci Rep.

